# Supplementary material for: Determining the educational impact of virtual patients on trauma team training during a multinational, large-scale civil military simulation exercise
Source: J Trauma Acute Care Surg. 2023 May 11;95(2):S99–S105. doi: 10.1097/TA.0000000000004017 (PMC10389465; doi:10.1097/TA.0000000000004017)
Supplement: Supplementary file 1 [file jt-95-s099-s001.docx]

**Determining the educational impact of virtual patients on major trauma team training during a multinational, large-scale civil military medical simulation exercise**

**Background**

Major trauma is any injury with the potential to cause prolonged disability or death and is now the leading cause of death and disability in the United States in those under 44 years old [1]. With asymmetrical conflicts, many countries now have an increasing number of major trauma events, including terrorist-associated incidents [1]. When these events occur, there is often limited capacity or the skills to deal with either the complex nature of the injuries or multiple patients. The nature of Western European medicine is currently characterised by early specialised training, more limited exposure to patients outside the chosen field, and limited exposure to trauma patients. Military style injuries now occur in a civilian environment. Many countries have reduced the size of their peacetime military medical capability making greater use of reserve personnel working in civilian hospitals, who have minimal exposure to the complex trauma patient, especially those with penetrating injuries or following explosive blast. Hospital based training comprises primarily of large-scale simulations but has neglected specific training on management of patients with complex, life threatening (sometimes competing) injuries or emphasising team skills [2, 3, 4].

Virtual patients are computer-based programs which present authentic cases and scenarios that simulate real-life scenarios. The participants function as healthcare providers is to obtain a history, conduct a physical examination, and make diagnostic and therapeutic decisions [5-9]. Although virtual patients have been used for decades in medical education, their design has been aimed at undergraduate students requiring binary decisions only. Virtual patients designed to be complex enough to challenge the reasoning and decision making skills of postgraduates, are still rare [10, 11]. There is an identified knowledge gap in the design of virtual patients with injuries and complexity similar to a real patient. Previous studies involved postgraduates who were experienced in their own (non-trauma) field but identified a gap in their decision-making when out of their professional context and zone of comfort. [11]. The team often understood an injury itself, but lacked the knowledge, and the experience to deal with competing injuries in the same patient, especially when there may be more than one way of addressing each one. The design of such virtual patients~~’~~ needs to reflect consequences, with further decisions required and choices to be made, to stimulate the decision making and team management of trauma patients.

Previous studies have also highlighted lack of knowledge and support for educators in surgical education, regarding integration of learning technologies [11, 12].  The existing learning technologies are mainly focusing on technical skills and there is a lack of learning technologies focusing on decision making and clinical reasoning [12, 13].

**Hypothesis:**

Assisted learning technologies such as virtual patients can augment simulated trauma team training and can improve trauma team competencies.

The aim of this study was to investigate whether virtual patients, developed from real-life patient data, integrated as a part of conventional trauma education, increased competencies in the decision making and team interaction required for the management of trauma patients during a multinational civil military trauma exercise, NATO Vigorous Warrior 2019.

**Objectives:**

- To determine the potential for a complex virtual patient to improve team interaction during simulated training
- To assess if a virtual patient model improves trauma competencies during simulated training
- To assess if a virtual patient model highlights competency gaps overall in the management of complex trauma patients, which can be addressed to improve care.
- To measure the acceptability of a virtual patient model in multinational civil military medical live simulation exercise

**Methods**

The study was an educational intervention prospective study where the population were exposed to the determination of novel virtual patient models, during a documented time period of 11 months: February-December 2019. The virtual patient models applied were interventions based on previous studies [12] and developed as branched clinical trauma scenarios focusing on non-technical skills and decision making in management of trauma patients with complex injuries, for example multiple gunshot wounds. The learner was required to make specific key management decisions based on choices offered. Each decision in turn, provided more decision choices affecting the outcome of the patient. Virtual patient models with a branched decision-making tree allow~~s~~ the learner to practice clinical reasoning and explore the management of the patient in depth, with specific good or poor outcomes on the patient, and encourages members of the team to interact, enhancing team communication.

The study design consisted of mixed methods, with multiple data collection methods applied to measuring the educational effects of virtual patients on trauma team training (Figure 1) [14, 15]. The study conforms to the STROBE Checklist of the EQUATOR Guidelines (Supplemental Digital Content, <http://links.lww.com/TA/D12>).

Figure 1. Reference diagram illustrating the study design

*Study Setting*

The project took place during a NATO multinational military exercise involving 18 participating nations, 16,000 troops including 1,600 moulaged "patients," across 10 Role 2 Field Hospitals, in Transylvania, Romania, from 4-9 April 2019. The hospitals were all tented or containerized, erected a day or two before the exercise commenced. A Standard Role 2 hospital is classified as having 7 Core modules available, including an initial immediate surgery response, and immediate post-operative care only. Enhanced modules (Role 2 Enhanced) such as Intensive Care or CT were not available. The Hospital Surgical Team contained a mix of surgeons, anaesthesiologists, emergency room physicians, nurses and paramedics deployed by their national military, and numbering a total 12-20 personnel. The concept of a "forward surgical team" is not consistent internationally - several teams were made up of a single surgeon, anaesthesiologist, emergency room physician, nurses and other emergency staff; many were very junior medical personnel, while others were highly professional and militarily experienced. Most were part-time military, working full-time in a variety of civilian healthcare facilities. Patient arrivals were often multiple, stretching the resources, and so disciplines were not as available for each patient, but spread across more than one. The patient load was consistently applied to the hospitals over five days, so staff may have been absent through rest periods and were diluted by the need for simultaneous care of several patients. There was a single Role 3 which was not part of our study. There was no equivalent of an ACS Level I (or even Level II Facility).

*Ethical Clearance*

Ethics approval was obtained from the supervising IRB: Nr: 2016/1701-31-5 Regional Ethical Review Board, Stockholm, Sweden and Nr: 12-15/2019 NATO Centre of Excellence for Military Medicine, Hungary.

With the assistance of the NATO Centre of Excellence for Military Medicine, participants were recruited between 1 December 2018 and 1 March 2019. Commanders of Military Field Hospitals representing several countries participating in the exercise, were responsible for communicating the study information and obtaining informed consent from the participants.

The data sources derived from: Individual pre- and post-assessments, evaluations made by experts in trauma education using the Team Emergency Assessment Measure protocol (TEAM) [16] when scoring performance during observations in combination with video-recordings of the trauma teams’ performance during the live simulation exercise [17-21].

*Inclusion and exclusion criteria*

The inclusion criteria for this study were: surgeons, anaesthesiologists, emergency department physicians, nurses, and operational paramedics or similar, all of whom were part of the deployment by their own National Military Service for the exercise. Many had only limited experience of joint civil military trauma care or austere conditions and needed to have adequate understanding of verbal as well as written English. The exclusion criteria were non-medical personnel or those with inadequate comprehension of English.

*Study population*

The population consisted of surgeons, anaesthesiologists, emergency room physicians, nurses and paramedics (n=30), made up six trauma teams, provided by trauma team members from eight countries (two teams used participants from two countries). Three trauma teams formed the participating group and three were allocated as the control group. were the participating group and three were the control group. The participating group were exposed to virtual patients pre- and post the live simulation exercise. One team of each group represented a full-time military team who had served in actual combat, one team consisted of senior experienced personnel, with some junior personnel, who were working predominantly in the civilian sector, and one group was made up of personnel with limited seniority and experience. The two groups had similar characteristics and differences in their makeup, seniority of the Team Leader, and breadth of training, but the control group had not participated in the virtual training.

*Data collection methods*

1. *Pre-assessment of experience in trauma management and use of learning technologies*

A virtual patient with a complex injury from a gunshot, with the focus on the decision-making required for management, was sent two months in advance to individuals in the participating group. The participants were asked to self-assess themselves on their own competence and skills [Appendix 1: Pre-assessment score Questionnaire - Supplemental Digital Content 1, <http://links.lww.com/TA/D13>].

2. *Individual assessment and evaluation of trauma management during exercise*

A similar virtual patient with increased complexity of gunshot injuries was provided individually to the participating group on 4-5th of April 2019, The results from the virtual patient and the gunshot case itself were then discussed during a seminar arranged by surgical experts in trauma education and training. The participants self-assessed themselves after the seminar.

3. *Evaluation of trauma teams’ performance during live simulation*

The participating group and the control group at military field hospitals received simulated trauma patients with gunshot and other competing injuries on 7-9 April 2019 as part of the exercise. The teams were expected to assess, resuscitate, and treat the patients. Team performance was assessed by independent observers who were experienced surgical trauma surgeons and educators [Appendix 2. Live exercise simulation: TEAM Score sheet - Supplemental Digital Content 2, <http://links.lww.com/TA/D14>]. Assessment included non-technical skills and interprofessional communication using video recordings, evaluation using the TEAM measurement scoring protocol [16], and assessment of clinical decision-making performance during the large-scale real-time simulation exercise. The performance of teams in the participating group were compared with teams in the control group [16].

4. *Individual post assessment of education and training in trauma management*

A further assessment was sent to the participating group 6 months after the live simulation exercise during December 2019, and participants were asked to self-assess their knowledge, performance, and experience in civil military trauma management [ Appendix 3. Post assessment score sheet. Questionnaire - Supplemental Digital Content 3, <http://links.lww.com/TA/D15>].They were also given the opportunity to provide written feedback (Figure 1).

*Analysis*

The Analysis was performed by the project team and consisted of discussions of results from each data set in relation to the hypothesis, aim and objectives of the study. In the prospective educational intervention study with applied mixed methods design, there were two different types of data sets to be analysed [14]. The first set of data was analysed, based on performance from individual and trauma teams’ assessments and evaluation of performance in the form of excel spreadsheets during the live simulation exercise. The second set of data evaluated the non-technical team skills from the live simulation by using the TEAM scoring protocol [16], with data sources from video recordings sessions, and the assessors’ observations and were analysed by applying the method of thematic analysis. Thematic analysis is defined as data analysis that involves the evaluation of specific themes and the identification of patterns within the data derived from the video recordings and observations. The thematic analysis consisted of six steps: 1. familiarizing with the data, 2. generating codes, 3. searching for themes, 4. reviewing themes, 5. defining and naming themes, 6. the final stage of a thematic analysis included the compilation of the themes in relation to the objectives and overall aim of the study [15, 17, 18, 19, 21].

This study was primarily aimed at a study group, who were exposed to virtual patient training before the exercise and compared to a control group. The research team had no control of the makeup of the trauma teams, nor roles of individuals in that team - all determined by their own military deployment. The assessment was based on level of knowledge displayed, and level of teamwork, and was based on a numerical scale of specific aspects of performance of both clinical and team traits [16]. The results are displayed as “splats” also known as “radar” diagrams, since the knowledge base affected the team interaction (and vice versa).

Patient outcomes were not assessed, since the assessment was from the point of arrival of the patient to the emergency area, where the assessment was done, and the assessment was stopped once the patient was moved to the next stage / tier of care. So, for example, a patient with head and abdominal injuries would have been moved out of the receiving bay for further care quite soon, and the assessment stopped. Outcomes of the care itself, apart from the initial decision making, cannot be determined. The nature of both the project, and the exercise, is that individual teams were not debriefed as they were fully occupied with other patients.

**Results**

Results from the data sources showed that virtual patient models were accepted by the participating group as a valid part of the simulation exercise during the training.

Data collected about the use of the virtual patients showed differences between the participating trauma teams (A-C) regarding time spent on the trauma scenarios presented by the virtual patients. On average, participants required two attempts to deal with virtual patient scenarios. Data from the virtual patients showed that most participants faced challenges at the same kind of key decision points, especially related to decision-making during examination of the injuries.

The trauma teams performances showed differences in their structures according to roles and responsibilities, experience, and national cultures. The participating group consisted of three field surgical teams from different NATO countries, each of which contained a mix of part time civilian-military and wholly military participants.

As displayed on the radar diagrams (Figures 2-4), each subject was scored from 0.1 (poor) to 1 (excellent).

Team A (Figure 2a) differed significantly from the other two teams by having exceptional knowledge and experience from actual combat experience and as full-time military medical personnel, although at various levels of seniority. Even this team reported virtual patients as valuable in stimulating their mindset by increased awareness of the different steps taken during the decision making process in managing trauma patients. There were no significant differences in Team A’s self-assessment before and after the exercise.

***Figure 2a***. Team A: Pre- and post- live simulation exercise

Team B (Figure 2b) was also made up of senior members but who were not used to working together as a team, as they were mainly not working in the military medical environment but worked in different civilian hospitals, had lower levels of existing knowledge and competence in managing major trauma patients and had needed to make more effective use of virtual patients. There was sometimes competition as to who was leading the team. It is of note that although their self-assessments (pre and post) were not significantly different from Team A, there was a trend towards over-confidence in the pre-assessment compared to the post-assessment.

***Figure 2b***. Team B: Pre- and post- live simulation exercise

Team C (Figure 2c). consisted of younger participants with much more limited experience or knowledge base for trauma, working almost exclusively in a peacetime civilian trauma environment, but who felt that there had been a substantial improvement in their knowledge and cooperation as a result of the simulation exercise.

***Figure 2c***. Team C: Pre- and post- live simulation exercise

During the subsequent live simulation exercise, the results from the evaluation conducted and scored by external trauma educational experts and the video recordings the teams’ performance, showed significant differences between the teams, in the management of major trauma patients due to different setups of roles and responsibilities, communication and delegation of work when receiving patients simultaneously (Figure 3).

***Figure 3***. Individual Team performance comparison during the live simulation exercise

Team A showed the best performance in the live simulations, with excellent scores in every aspect of the assessment. This is not unexpected in a team who were experienced in the care of the complex trauma patient, and who were used to regularly working with each other.

Team B, with experienced senior members, also scored well, but the assessment showed areas which could have been improved upon, particularly in the assessment of the live simulated patient, and there was a “top down” approach, rather than “bi-directional interaction”, between the senior and junior members of the team, so that aspects of patient injuries and care were omitted as some vital data and information were not communicated between the team leader, and the less experienced members, and therefore prioritization of care suffered.

Team C were younger, with a lower baseline of knowledge and experience, so they had more difficulty in adapting to the “changing” state of the patient, and each member concentrated on their own tasks, without the necessary coordination nor communication between team members.

The comparison of the performance of the participating groups compared to the matching control groups showed improved performance in favour of the groups exposed to virtual patients. (Figure 4) Both groups showed weaknesses in the documentation of the care, and some differences in the overall assessment which would be suitable aims for education of subsequent teams.

***Figure 4.*** Participating group compared to the control group during the live simulation exercise

The effective utilization of virtual patients as support for reasoning, during the learning process in decision making, was negatively correlated to levels of previous knowledge and experience. Using virtual patients contributed to improved individual knowledge and decision making within conventional teaching. Analysis also showed improved interprofessional teamwork for professionals less experienced in military medicine or with working in austere environments.

The analysis of qualitative data from the individual pre-assessments, video recorded observations during the educational trauma seminar as well as post-assessments, suggested increased awareness of flexible thinking, individually and within the teams.

**Discussion**

This study was an educational interventional prospective study with mixed method design, where the population were exposed to the determination of novel virtual patient models, over a documented time period of 11 months. The multiple data collection methods applied to measuring the educational effects of virtual patients on trauma team training. However, the purpose with mixed methods was to explore and investigate how the virtual patient model was received, if it effected and improved non-technical skills in the management of major trauma patients, and whether there were any gaps in competency which could be identified and indicators for evaluation of educational techniques to improve clinical performance of civil military trauma teams in the future.

The results confirmed variability that, in what should have been similar training, differed across a wide organization, with many languages, national traits, and variety, both in the training itself, but also in the case mix seen by the professionals in their non-military jobs.

All moulaged patient scenarios were assessed by at least two trauma surgeons, who were trained surgical educators and experienced in the Non-Technical Skills Program. Trauma patients presented virtually, and in the assessment and management of cases such as were presented during the exercise. Unfortunately, blurring took place in trauma teams, where the non-technical skills and knowledge base were dependent on each other, and weakness in one negatively affected the other.

Identified significant indicators (Figure 3) represented areas of potential as support for improvement in major trauma education and training. The indicators are generic and applicable in the design of education and training aimed for this target group. Team B, with experienced team leaders, highlighted gaps in two-way interaction which could be addressed. The most inexperienced trauma team (Team C) had the greatest potential for change, and educational policies which are modified to take this into account. The identified indicators as results from this study are valuable as a basis for the design of future studies and will be used in planning of the next multinational civil military medical exercise scheduled for 2024.

Different set ups of roles and responsibilities between the trauma teams affected the quality of data and challenges in analysis of results from data sources. Extended inclusion and exclusion criteria for future studies are of importance and standardised templates need~~s~~ to be developed.

 Differences in the level of experience of military medicine or work in austere environments showed significant differences in learning outcomes for the trauma teams who had less experience as compared to the teams with more experience of dealing with combat injuries. This could have been related both to the maturity of each team, and the ease of communication between team members. Improved individual knowledge and interprofessional teamwork were directly correlated to utilizing virtual patients in combination with conventional teaching of managing military trauma patients and is most efficient for the less experienced group. This kind of indicator is helpful in the future design of education and training as well as development of suitable virtual patients.

Of particular interest is that the most inexperienced participant team (Team C), showed the greatest improvement as their confidence improved, both in their skills and their approach to the critically ill patient.

The educators’ levels of experience about how to integrate learning technologies in teaching and training to support learning and provide flexibility for participants is of great importance since the educators as senior trauma professionals have an enormous impact on the education and training system. Previous studies have highlighted needs for new educational methods and development of new learning technologies for postgraduates. Virtual patients aimed for the target group have been validated as results from this study, and provide the support for educators’ understanding to integrate learning technologies when designing education and training. Virtual patient models are also able to provide flexible learning and training, highlighted in previous studies and validated during this study.

**Conclusion**

The results show a trend which might reduce the actual time spent, and the costs involved in trauma education, by allowing more time to be available focused on practical skills training and teamwork. The use of virtual patients to change the mindset and philosophy of participants before they attend the course or training exercise, seems to have great potential. The results demonstrated several advantages in the use of virtual patients as preparation for major trauma exercises and appear to be supportive, especially for teams with limited experience. The key to success is however the support for educators, to increase their knowledge of how to integrate learning technologies because of their impact in the education and training system. Identified indicators as part of the results might be the basis for future design of modern military medical education and training.

This educational study was helpful in identifying and designing methods for both medical and team training, by highlighting limitations in the application of combat care. Many combat teams learn their skills in time of conflict and there is a major attrition of these in times of peace.

The results highlighted different areas for improvement, which were themselves different, not only on the knowledge and confidence base, but also the seniority and team interaction between the various members of the same team. Learning technologies such as virtual patients in combination with more conventional teaching and simulation methods supported preparation prior to the exercise and indicated the design of future virtual patients, both for better care, and for better integration within the trauma team. The lessons from the study will be applied in a prospective interventional study focusing on evaluation of educational techniques to improve performance of civil military trauma teams, and with significant larger number of participants, planned for the next major exercise scheduled for 2024.

**Supplemental Digital Content**

Supplemental Digital Content 1: Appendix 1:  Pre-assessment score Questionnaire

Self-administered Questionnaire, based on the first virtual patient, answered and submitted before the exercise

Supplemental Digital Content 2: Appendix 2.  Live exercise simulation: TEAM Score sheet

TEAM score sheet used by Expert Trauma Educators during the live simulation

Supplemental Digital Content 3: Appendix 3.  Post assessment score sheet. Questionnaire

Self-administered Questionnaire answered and submitted 6 months after the exercise.

*Statements of author contribution, declaration of conflict of interests and funding*

The authors meet ICMJE requirements for authorship and declare no conflict of interests.  All authors have made substantial contributions to the following: (1) the conception and design of the study, analysis, and interpretation of data, (2) drafting the article and revising it critically for important intellectual content, (3) final approval of the version to be submitted.

There is no external funding for the study.

**References**

1. National Centre for Injury Prevention and Control (NCIPC). Injuries and Violence Are Leading Causes of Death. Available at: https://www.cdc.gov/injury/wisqars/animated-leading-causes.html. Centres for Disease Control and Prevention. Atlanta, GA. Accessed January 25, 2023.
2. Sonesson L, Boffard K, Lundberg L, Rydmark M, Karlgren K. The potential of blended learning in education and training for advanced civilian and military trauma care. *Injury.* 2018; 49:93-96.
3. Escher C, Rystedt H, Creutzfeldt J, Meurling L, Nyström S, Dahlberg J, et al. Method matters: Impact of in-scenario instruction on simulation-based team training. *Advances in Simulation (Lond).* 2017; 2:25.
4. Wisborg T, Brattebø G, Brinchmann-Hansen A, Uggen PE, Hansen KS. Effects of nationwide training of multiprofessional trauma teams in Norwegian hospitals. *J Trauma.* 2008; 64:1613-8.
5. Ellaway R, Poulton T, Fors U, McGee JB, Albright S. Building a virtual patient commons. Med Teach 2008; 30:170-174.
6. Kononowicz A. A, Zary N, Edelbring S, Corral J, Hege I. Virtual patients-what are we talking about? A framework to classify the meanings of the term in healthcare education. *BMC Med Educ* 2015; 15:11.
7. Edelbring S. Technology in education, necessary but not sufficient: understanding learning with virtual patients. Karolinska Institutet, Management and Ethics. Stockholm: Karolinska Institutet; 2012.
8. Ellaway R, Toops D, Lee S, Armson H. Virtual patient activity patterns for clinical learning. *Clin Teach*. 2015; 12:267-71.
9. Rystedt H, Lindström B. Introducing simulation technologies in nurse education: a nursing practice perspective. *Nurse Education in Practice.* 2001; 1:134-41.
10. Ekblad S, Mollica RF, Fors U, Pantziaras I, Lavelle J. Educational potential of a virtual patient system for caring for traumatized patients in primary care. *BMC Medical Education* 2013; 13:110.
11. Sonesson L, Boffard KD, Lundberg L, Rydmark M, Karlgren K. Decision-making in management of the complex trauma patient: Changing the mindset of the non-trauma surgeon. *World J Surg*. 2018; 42:2392-2397.
12. Sonesson, L. 2018. *Design of blended learning for civilian and military trauma care.* Karolinska Institutet, Stockholm, Sweden. Eprint AB. ISBN: 978-91-7676-955-3.
13. Murray DJ, Freeman BD, Boulet JR, Woodhouse J, Fehr JJ, Klingensmith ME. Decision making in trauma settings: simulation to improve diagnostic skills. *Simul Health*, 2015;10:139-45.
14. Onwuegbuzie, Anthony J., Hitchcock, John H. 2022. *The Routledge handbook for advancing integration in mixed methods research*. London: Routledge.
15. Mor Y, Winters N.  Design approaches in technology-enhanced learning*.* *Interactive Learning Environments*. 2007; 15:61-75.
16. Cooper S, Cant R, Porter J, Sellick K, Somers G, Kinsman L, et al. Rating Medical Emergency Teamwork Performance: Development of the Team Emergency Assessment Measure (TEAM). *Resuscitation*. 2010; 81:446-52.
17. Heath C, Hindmarsh J, Luff P. *Video in Qualitative Research*. 2010, London: SAGE.
18. Braun V, Clarke V. Using thematic analysis in psychology. Qualitative Research in Psychology, 2006; 3:77-101.
19. Derry SJ, Pea RD, Barron B, Engle RA, Erickson F, Goldman R, et al. Conducting video research in the learning sciences: Guidance on selection, analysis, technology, and ethics. *Journal of the Learning Sciences*. 2010; 19**:**3-53.
20. World Medical Association: WMA Declaration of Helsinki – ethical principles for medical research involving human subjects. Available from https://www.wma.net/policies-post/wma-declaration-of-helsinki-ethical-principles-for-medical-research-involving-human-subjects/ . (Accessed 28 January 2020).
21. Bezemer J, Cope A, Korkiakangas T, Kress G, Murtagh G, Weldon SM, et al. Microanalysis of video from the operating room: An underused approach to patient safety research. *BMJ Quality and Safety.* 2017; 26:583-587.

**Legends for figures**

**Figure 1**. Reference diagram illustrating the study design

***Figure 2a***. Team A: Pre- and post- live simulation exercise

***Figure 2b***. Team B: Pre- and post- live simulation exercise

***Figure 2c***. Team C: Pre- and post- live simulation exercise

***Figure 3***. Individual Team performance comparison during the live simulation exercise

***Figure 4.*** Participating group compared to the control group during the live simulation exercise
